# Alkylated EDTA potentiates antibacterial photodynamic activity of protoporphyrin

**DOI:** 10.1186/s12951-024-02353-3

**Published:** 2024-04-08

**Authors:** Ying Piao, Sebastian Himbert, Zifan Li, Jun Liu, Zhihao Zhao, Huahai Yu, Shuangshuang Liu, Shiqun Shao, Michael Fefer, Maikel C. Rheinstädter, Youqing Shen

**Affiliations:** 1 Zhejiang Key Laboratory of Smart Biomaterials and Key Laboratory of Biomass Chemical Engineering of Ministry of Education, College of Chemical and Biological Engineering, Zhejiang University, Hangzhou, 310058 China; 2 Department of Physics and Astronomy, McMaster University, Hamilton, ON L8S 3Z5 Canada; 3 Suncor AgroScience, Mississauga, ON L5K 1A8 Canada

## Abstract

Antibiotic resistance has garnered significant attention due to the scarcity of new antibiotics in development. Protoporphyrin IX (PpIX)-mediated photodynamic therapy shows promise as a novel antibacterial strategy, serving as an alternative to antibiotics. However, the poor solubility of PpIX and its tendency to aggregate greatly hinder its photodynamic efficacy. In this study, we demonstrate that alkylated EDTA derivatives (aEDTA), particularly C14-EDTA, can enhance the solubility of PpIX by facilitating its dispersion in aqueous solutions. The combination of C14-EDTA and PpIX exhibits potent antibacterial activity against *Staphylococcus aureus* (*S. aureus*) when exposed to LED light irradiation. Furthermore, this combination effectively eradicates *S. aureus* biofilms, which are known to be strongly resistant to antibiotics, and demonstrates high therapeutic efficacy in an animal model of infected ulcers. Mechanistic studies reveal that C14-EDTA can disrupt PpIX crystallization, increase bacterial membrane permeability and sequester divalent cations, thereby improving the accumulation of PpIX in bacteria. This, in turn, enhances reactive oxygen species (ROS) production and the antibacterial photodynamic activity. Overall, this effective strategy holds great promise in combating antibiotic-resistant strains.

**Graphical Abstract:**

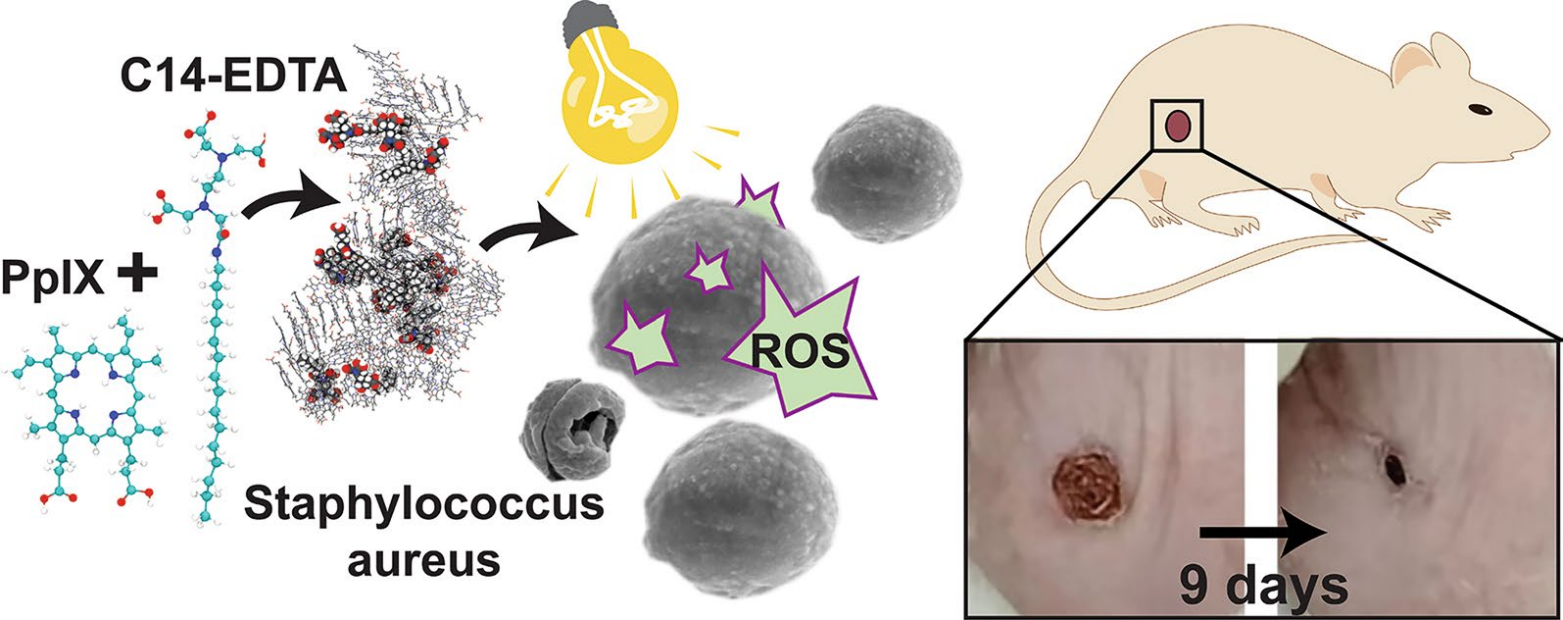

**Supplementary Information:**

The online version contains supplementary material available at 10.1186/s12951-024-02353-3.

## Introduction

Antibiotic resistance is a significant global public health threat, resulting in at least 1.27 million deaths worldwide in 2019 [1]. The misuse and overuse of antibiotics are the primary factors driving the development of drug-resistant strains [2, 3]. Despite the urgent need for new antibiotic therapies, the pace of antibiotic discoveries has noticeably slowed down in recent decades due to scientific challenges and inadequate financial incentives [4]. This necessitates the exploration of innovative approaches to combat bacteria using mechanisms distinct from traditional antibiotics.

Photodynamic therapy (PDT) stands as a promising alternative to traditional antibiotics. The antimicrobial effect of PDT relies on a photosensitizer [5, 6], which can be activated by light to generate reactive oxygen species (ROS), such as hydroxyl radicals (type I) or singlet oxygen (type II). These ROS indiscriminately inactivate bacteria through an oxidative burst [7]. Due to the high reactivity of ROS and the vulnerability of intracellular components to oxidation, bacteria have limited capacity to counteract ROS, suggesting that repeated photosensitization may not lead to drug resistance [8]. Protoporphyrin (PpIX) is a commonly used photosensitizer due to its excellent biocompatibility [9]. However, its poor water solubility and tendency to aggregate hamper efficient singlet oxygen production, thereby limiting PDT efficacy [10]. Previous attempts to overcome these limitations involved concerting PpIX into derivatives or fabricating nanoparticles, but these strategies were often prohibitively complex and achieved limited success in eliminating bacteria pathogens [11–17]. Identifying an enhancer to potentiate PpIX-mediated PDT could offer a more effective and direct solution to tackle this challenge.

Ethylenediaminetetraacetic acid (EDTA) can chelate and thus deprive of the divalent cations, such as Mg^2+^ and Ca^2+^, from bacterial outer layer walls and biofilms, affecting bacterial membrane permeability and demonstrating mild antimicrobial and antibiofilm activities [18]. EDTA has been shown to enhance the efficacy of other antimicrobial agents [19, 20]. For instance, in the treatment of resistant bacterial biofilms in diabetic foot infections, EDTA synergized with indocyanine green to enhance PDT activity. However, this synergistic effect was relatively weak, 5 mM EDTA resulting in less than a tenfold reduction in bacterial viability [21]. EDTA is water-soluble so EDTA introduced with a long alkyl chain would bind bacterial membrane and thus more effectively enhance the antibacterial activity of antibacterial agents. Indeed, alkylated EDTA with fatty chains loaded in self-emulsifying drug delivery systems displayed twofold higher degrees of antimicrobial activity compared to EDTA [22, 23].

In this study, we synthesized a series of alkylated EDTA derivatives with different alkyl chains. These alkylated EDTAs (aEDTAs), particularly EDTA mono-tetradecylamide (C14-EDTA), effectively reduced the aggregation of protoporphyrin (PpIX), facilitated its accumulation in bacteria, and catalyzed singlet oxygen production, significantly enhancing PpIX’s antimicrobial and biofilm eradication activities. We speculate that the PDT-enhancing activity of C14-EDTA may be attributed to its synergy of dispersing PpIX and anchoring onto and destabilizing the bacterial outer membrane.

## Materials and methods

### Materials

Protoporphyrin, ethylenediaminetetraacetic dianhydride (EDTAD), ethylenediaminetetraacetic acid (EDTA), octylamine, 1-dodecanamine, 1-aminotetradecane, 1-aminopentadecanne, 1-hexadecylamine, 1-octadecylamine were purchased from Leyan Chemical (Shanghai, China). Lysogeny broth medium (LB medium), nutrient agar medium, DCFH-DA were purchased from Sigma-Aldrich. PpIX was purchased from Macklin. All the organic solvents otherwise indicated were purchased from Sinopharm Chemical Reagent Co., Ltd. (Shanghai, China).

### Instruments and software

The ^1^H-NMR spectra of all chemical intermediates and products were recorded using a Bruker Avance DRX-400 spectrometer (400 MHz) with CDCl_3_ or DMSO-d6 as solvent. The particle size was measured using a Malvern Nano ZS Zetasizer (Malvern Instrument Ltd., UK) with He–Ne laser light (632.8 nm) at 173° scattering angle at 25 °C. The volume-averaged diameter was obtained from the instrument's Dispersion Technology Software version 6.1. The aggregation of PpIX was observed using a scanning electron microscope (Hitachi, SU8010). ROS value was evaluated by Flow Cytometry (BD, FACS Calibur). The live-dead fluorescence staining of biofilms was observed by Confocal Laser Scanning Microscopy (Nikon A1) with 488 nm and 543 nm excitation laser light.

### Synthesis of alkylated EDTA derivatives

**Scheme 1.**
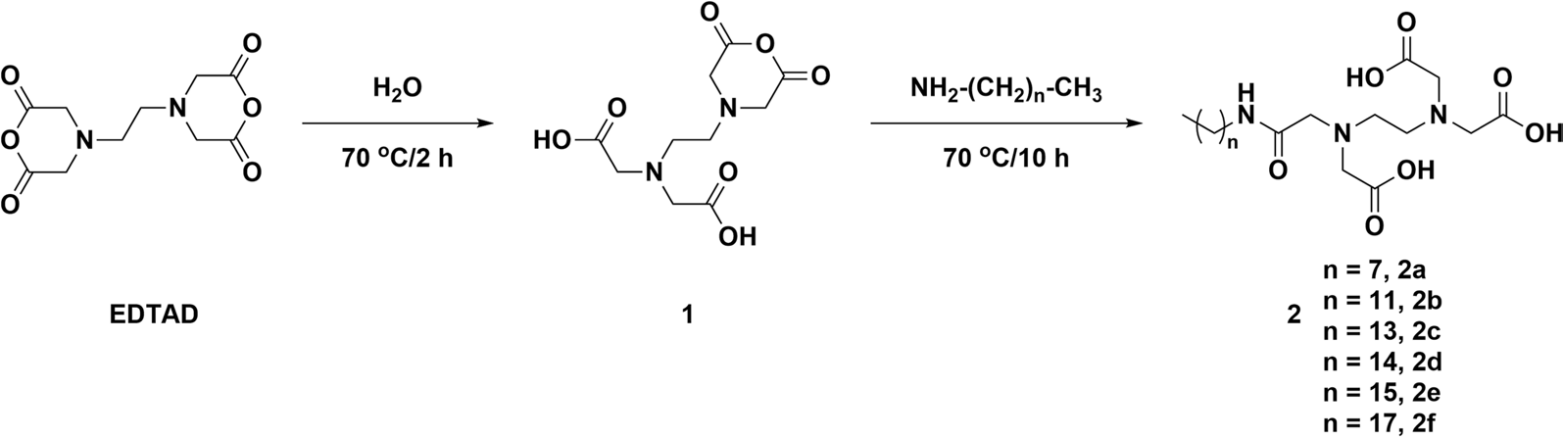
General synthetic route to aEDTA

Synthesis of alkylated EDTA derivatives was performed as previously reported from EDTA dianhydrides (EDTAD) [24] and is sketched in Scheme 1. EDTAD (16.73 g, 65.3 mmol) was dissolved in dry DMF (80 mL) in a round-bottom flask at 70 °C. Then water (0.94 mL, 52.2 mmol) diluted in 10 mL of dry DMF was dropwise into the flask in 30 min. Subsequently, the reaction mixture was stirred at 70 °C for 2 h and cooled to room temperature to give ethylenediaminetetraacetic monoanhydride (intermediate 1) as a precipitate. The solid was filtered and washed with 50 mL of dry DMF and dried under vacuum. The intermediate 1 (17.92 g, 65.3 mmol) and alkylamine (58.8 mmol) were added into 70 mL of dry DMF and stirred at 70 °C for 10 h. Afterwards, the reaction mixture was cooled to room temperature and poured into water (400 mL). The precipitate was collected by centrifugation and washed with 50 mL of water. The product was gained after lyophilization. All EDTA derivatives (2) were validated using the quadrupole time-of-flight mass spectrometry (qTOF-MS) (Additional file 1: Figures S1–S6).

### Bacterial strains

*Staphylococcus aureus* (*S. aureus*) was purchased from China General Microbiological Culture Collection Center. Sequence analysis showed that it was derived from the *S. aureus* strain ATCC-12600, with an accuracy rate of identification at 99.57%. Bacteria were grown on Luria–Bertani (LB) agar medium.

### Photosensitizer and illuminator

A stock solution of PpIX (1.0 mg mL^−1^, equal to 1.78 mM) was prepared by dissolving PpIX into the NaOH PBS solution at pH 9.0. The solution was stored at 4 ℃ in the dark. The illuminator was purchased from Youke Instrument & Equipment Corporation, equipped with an LED light source covering the wavelength range from 380 to 780 nm and an extra 640 nm red light (Additional file 1: Figure S7). The distance between the light source and the sample position was 30 cm, and the maximum illumination intensity at this position was ~ 90 W/m^2^. For all experiments conducted under illumination, the maximum intensity of the illuminator was used.

### Bacterial viability assay for different drug combinations

A suspension of *S. aureus* was added to each well of a 96-well plate at a density of ~ 1 × 10^8^ (colony-forming unit) CFU in 80 μL per well. Subsequently, 10 μL of PpIX and 10 μL of either EDTA or aEDTAs were added to the wells. The final concentrations of PpIX were 5, 10, and 100 μg mL^−1^ (equal to 8.89, 17.77, 177.73 μM), respectively. The concentrations of EDTA and aEDTAs were 0.5 mM and 0.05 mM, respectively. The plate was incubated in the dark for 1 h at room temperature and then illuminated for 1, 2, or 3 h using the illuminator described above. A broth dilution method was used to test the antimicrobial activities of the drugs. Specifically, the bacterial solution of each well was serial diluted by 10 to 10^5^ times with PBS and then 10 μL of the suspension in each well was transferred to the LB agarose plates. The bacteria amount was counted after incubation at 37 °C for 24 h.

### Biofilm eradication assay

The crystal violet assay was employed to evaluate the biofilm eradication efficacy of the compounds. Briefly, an exponentially growing suspension of *S. aureus* (1 × 10^9^ CFU mL^−1^) was added to each well of a 96-well plate at 200 μL per well. The plate was incubated at 37 °C for 24 h to allow the formation of a mature biofilm. Subsequently, the wells were rinsed three times with saline to remove the unbound planktonic bacteria. Solutions containing 17.77 μM of PpIX and various concentrations of EDTA or C14-EDTA (0.05, 0.1, or 0.5 mM) were added to the biofilms. After incubation at 37 °C for 24 h, the biofilm was illuminated for 1 h or 2 h. Then, the supernatant was removed and the biofilm residue was rinsed gently with saline three times. Afterward, 80 μL of 95% ethanol was added to each well and incubated for 15 min to fix the samples, followed by adding 0.1% crystal violet for another 15 min to allow the crystal violet to adhere to the proteins in the residual bacteria. The medium containing crystal violet was then removed and washed three times with PBS. Finally, 120 μL of 33% acetic acid was added to each well to solubilize the crystal violet and the absorbance in each well was measured at 560 nm. The biofilm viability relative to the untreated group was then calculated.

### Biofilm live/dead staining assay

Mature biofilms were formed by incubating 1 × 10^7^ CFU of bacteria on a sterile confocal plate for 24 h. After washing with saline three times, the biofilms were incubated with PpIX (17.77 μM) alone or its combination with 0.1 mM of EDTA/C14-EDTA for 24 h and then illuminated for 1, 2, or 3 h. The residual biofilms were washed with saline, stained with Syto9 (10 μg mL^−1^) and propidium iodide (PI, 10 μg mL^−1^) for 15 min, and then examined using a confocal laser scanning microscopy (CLSM) at (Ex/Em) 488 nm/500 nm for Syto9 and 543 nm/635 nm for PI.

### ROS detection

Exponentially growing *S. aureus* were seeded to each well of a 24-well plate at a density of ~ 1 × 10^9^ CFU per well. PpIX (17.77 μM) or PpIX with EDTA (0.05 mM) or C14-EDTA (0.05 mM) was added into each well in a volume of 500 μL. After incubation in the dark for 1 h, 2ʹ,7ʹ-dichlorodihydrofluorescein diacetate (DCFH-DA) (0.01 mM) was added into each well and incubated for an additional 15 min at room temperature. The plate was then illuminated for 30 min, and the suspension in each well was transferred to separate tubes. The bacteria in each tube were collected by centrifugation and washed with saline three times. The washed bacteria were then dispersed in 0.2 mL of PBS and the fluorescent intensity of the bacteria was measured using flow cytometry (Ex: 488 nm; Em: 525 nm).

### Scanning electron microscope observation

The particles of PpIX or its combination with EDTA or C14-EDTA were observed by a field emission scanning electron microscope (SEM) (Hitachi, SU8010, Japan) operating at 3.0 kV. The samples were prepared by casting a drop of PpIX dispersion onto a 200-mesh copper grid without filtration. Then, a gold film was coated on the sample and allowed to air-dry before observing the sample under the microscope.

### Animal model of infected ulcers

All animals were purchased from Shanghai SLAC Laboratory Animal Co., Ltd. and housed in a specific pathogen-free (SPF) animal facility at Zhejiang University. All animal experiments were carried out under the protocols approved by the Institutional Animal Care and Use Committee (IACUC) of Zhejiang University in accordance with the institutional guidelines.

Wounds were created on the skin of BALB/c mice (female, 8–10 weeks old, 18–22 g) with an 8 mm diameter punch. Methotrexate (10 mg kg^−1^) was intraperitoneally injected to the mice. The injections were given one day before the wound induction and two days after the wound induction. A suspension of *S. aureus* (1.5 × 10^7^ CFU in 20 μL) was inoculated into the wound sites to allow the formation of mature biofilms at the wound sites. Sterile dressings were then applied to cover the ulcers. Treatments were performed on day 3, 5, and 7 by immersing the ulcer regions into the solution of PpIX and EDTA or C14-EDTA for 1 h, followed by illumination for 2 h. After each treatment, a sterile miniswab was tightly covered on the ulcer regions for 20 min. The miniswab was immersed in 1 mL of saline and sonicated for 2 min to detach the bacteria into the solution. The amount of bacteria was determined using the standard plate count method.

### Molecular Dynamics simulations

Molecular Dynamics (MD) simulations were performed on a GPU-accelerated workstation using GROMACS Version 2022.3. All atom models of PpIX, C14-EDTA and C8-EDTA were designed using the CHARMM-GUI ligand-modeler (http://charmm-gui.org/) [25] and the CHARMM36 force-field [26]. Three systems were designed using the CHARMM-GUI multicomponent builder, as listed in Table 1. A system containing 100 PpIX molecules was run for 100 ns. 14 C14-EDTA, 14 C8-EDTA or 14 EDTA molecules were subsequently added to the system and the system was allowed to run for an additional 200 ns, respectively. All simulations were minimized and equilibrated for 5 ns in the NPT ensemble (constant pressure and temperature), at T = 37 °C. The simulation used a 1 fs time step, a short-range van der Waal cutoff of 1.1 nm and a potential-shift-Verlet coulomb modifier. Periodic boundary conditions were applied to all spatial directions. Neighbor lists were updated in intervals of 20 steps. The temperature coupling was controlled by a *v*-rescale thermostat at a constant pressure of 1 bar using Parrinello-Rahman semi-isotropic weak coupling (τ = 12 ps; compressibility β = 3·10^–4^ bar^−1^).

**Table 1:** Composition of the four simulated all-atom and two Martini coarse grained simulations

Component	PpIX (# of molecules)	C14-EDTA (# of molecules)	C8-EDTA (# of molecules)	EDTA (# of molecules)
All-atom simulation				
PpIX-AA	100	0	0	0
PpIX-C14-AA	100	14	0	0
PpIX-C8-AA	100	0	14	0
PpIX-EDTA-AA	100	0	0	14
Martini simulation				
PpIX-CG	2000	0	0	0
PpIX-C14-CG	2000	2000	0	0

The all-atom simulations were supplemented by coarse grained simulations using the Martini force-field. [27] First, coarse-grained models of PpIX and C14-EDTA were created. Previously mapped hemoglobin topologies were used in the design of PpX. The porphine core was taken from the hemoglobin model [28] while the iron core was removed without supplement. C14-EDTA did not exist in the Martini force-field. Consequently, the model was parametrized using common practices. Briefly, an all-atom model of a single C14-EDTA molecule was simulated for 200 ns. Afterwards the trajectory was mapped according to the designed theme in the Additional file Material (Additional file 1: Fig. S14). The distribution of the bond length and bond angle for every molecular bond were measured from the all-atom trajectory. Afterwards, initial force-field parameters were defined, and a single coarse-grained molecule was simulated for 200 ns. The distributions of bond lengths and angles were qualitatively compared to the all-atom results. The force-field parameters were then adjusted, and the simulation was allowed to rerun. This process was repeated until the distributions of bond-length and bond-angle showed sufficient agreement. The bond length and bond angle distributions of the final model are visualized in the Additional file Material (Additional file 1: Figure S14). The force-field parameter of the all-atom and coarse–grained models are available as Additional files (Additional file 1: Figure S15).

### Statistical analysis

All experiments were performed in triplicate. Quantitative data were presented as means ± standard deviation (SD). Student’s t-tests were performed to analyze the significance of differences between the two groups. The difference was considered to be significant when *P < 0.05, **P < 0.01, ***P < 0.001, and ****P < 0.0001.

## Results and discussion

### The effect of aggregation on the antibacterial photodynamic activity of PpIX

**Fig. 1:**
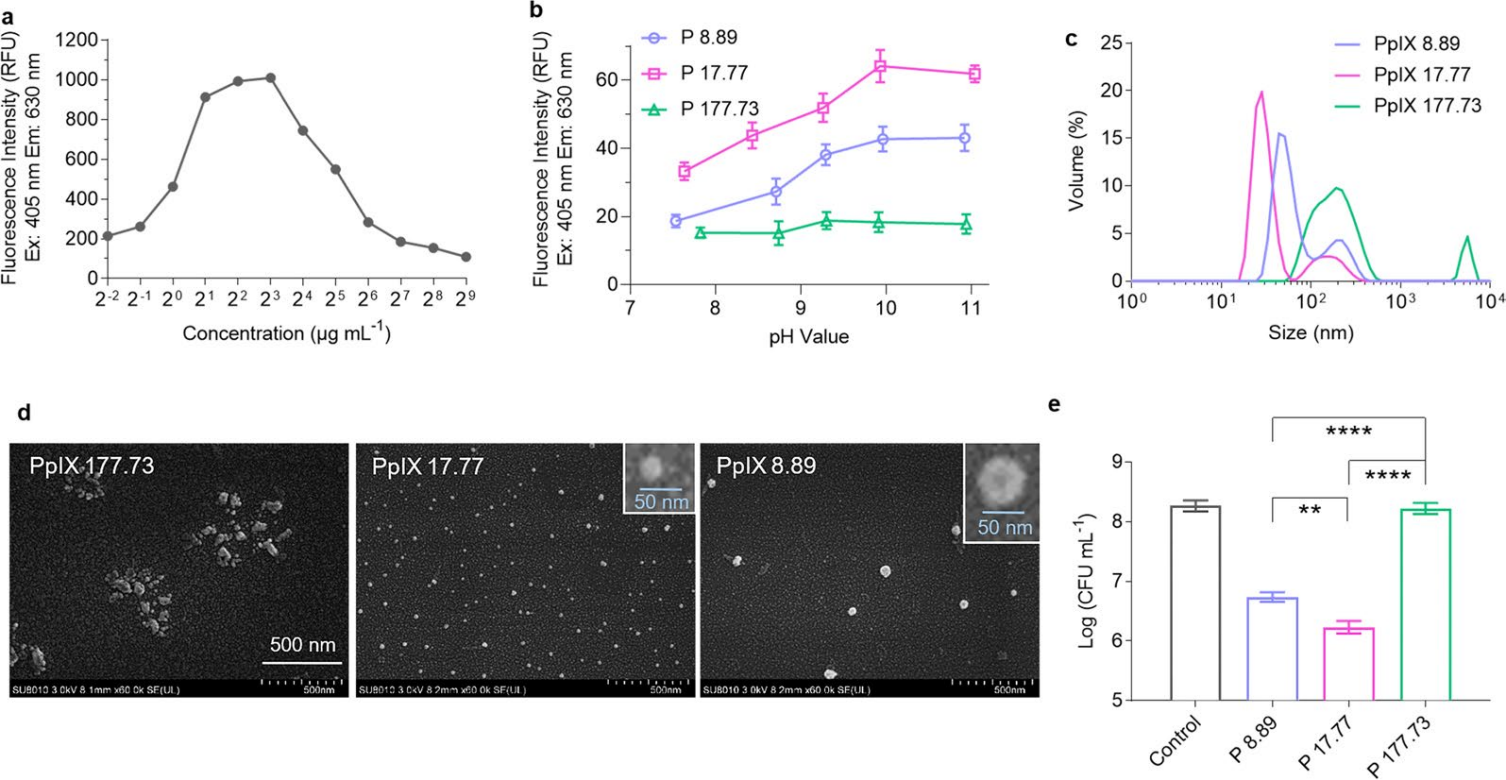
Properties of PpIX at different concentrations. **a** Fluorescence intensity of PpIX at different concentrations. **b** Fluorescence intensity of PpIX at three representative concentrations in aqueous solutions at different pH values. **c** Size distribution of PpIX formed at three different concentrations (pH 10.0) measured by DLS. **d** SEM images of the PpIX particles. **e** Antibacterial activity of PpIX at the three concentrations against *S. aureus* after 2 h illumination; Data are presented as mean ± SD (*n* = 3). **P < 0.01, ****P < 0.0001

Generally, a photosensitizer would have a weak fluorescence if it produces singlet oxygen effectively. Nonetheless, for PpIX, the poor water solubility and readily aggregation quench its fluorescence as well as singlet oxygen production. This permits us to investigate the concentration-dependent aggregation of PpIX by detecting the fluorescent emission. We first measured the fluorescence intensity of PpIX at different concentrations in DMSO, which peaked at approximately 8 ~ 10 μg mL^−1^ (Fig. 1a). We selected three concentrations around 10 μg mL^−1^ (5, 10, and 100 μg mL^−1^, equal to 8.89, 17.77, and 177.73 μM, respectively). As pH conditions can affect the PpIX’s solubility and hence fluorescence, we determined the pH-related fluorescence intensity of PpIX in aqueous solutions to reflect the aggregation (Fig. 1b). The PpIX solution at 17.77 μM consistently had stronger fluorescence than that at 8.89 μM or 177.73 μM. The DLS measurements showed that PpIX at 17.77 μM exhibited a primary peak at 30 nm and a small secondary peak at 150 nm, while the solution at 177.73 μM had larger particles and even some aggregates of up to 5500 nm (Fig. 1c). These findings were further confirmed by SEM imaging (Fig. 1d). Surprisingly, PpIX at 8.89 μM formed 50 nm particles for an unknown reason. So, the fluorescence intensity of PpIX was consistent with its formed particle sizes.

Next, we evaluated the antibacterial photodynamic activity of PpIX against planktonic *S. aureus*, a Gram-positive microbial pathogen, at the three concentrations mentioned above. After illuminated for 2 h, PpIX at 17.77 μM or 8.89 μM reduced the bacterium population by 2 or 1.5 logs, respectively, whereas 177.73 μM PpIX did not affect bacterial proliferation (Fig. 1e). This result is consistent with the concentration-dependent aggregation and activity in the photodynamic inactivation of PpIX.

### aEDTA-enhanced PpIX photodynamic activity against *S. aureus*

**Fig. 2:**
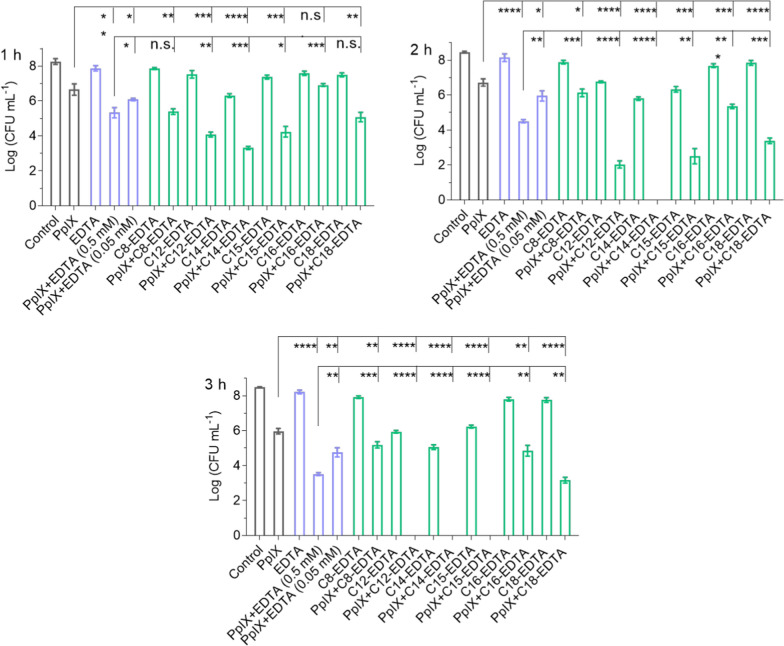
aEDTA improved PDT efficacy of PpIX against planktonic *S. aureus*. Exponentially growing *S. aureus* were preincubated separately with the combinations of PpIX (17.77 μM) and EDTA (0.5 mM) or aEDTAs (C8-EDTA, C12-EDTA, C14-EDTA, C15-EDTA, C16-EDTA, or C18-EDTA; 0.05 mM EDTA-eq.) for 1 h followed by illumination for 1 h, 2 h, or 3 h. The bacterium viability was determined by the standard plate count method; Data are presented as mean ± SD (*n* = 3). ****P < 0.0001, ***P < 0.001, **P < 0.01, *P < 0.1

The PpIX’s photodynamic activity (PDT) in the presence of aEDTAs with alkyl chains of varying lengths (C8, C12, C14, C15, C16, and C18) was evaluated. PpIX (17.77 μM) with EDTA or aEDTAs was cultured with *S. aureus* (~ 1 × 10^9^ CFU mL^−1^) for 1 h followed by illumination for 1, 2, or 3 h. As shown in Fig. 2, the sensitized PpIX PDT activity was determined by the illumination time and EDTA types. Longer illumination facilitated the bacteria-killing. For instance, 1, 2, or 3 h illumination of PpIX with 0.05 mM C12-EDTA reduced the CFU by 4, 6 or greater than 8 log. A 1 h illumination of all the combinations did not eliminate the bacteria, resulting in a maximum 5 log CFU reduction, a 2 h illumination of PpIX with 0.05 mM C14-EDTA eradicated the bacteria. In addition, compared with EDTA, aEDTAs alone showed relatively higher antibacterial activity, which can be possibly attributed to two aspects. First, aEDTAs can insert into the bacterial membrane and damage the membrane integrity. Second, aEDTAs can chelate bivalent ions such as Ca^2+^, which can also contribute to the antimicrobial effect.

Figure 2 also shows that aEDTAs were significantly more effective in enhancing the PDT activity of PpIX. For instance, PpIX alone reduced the bacterium CFU by 2 logs after 3 h illumination and its combination with 0.05 or 0.5 mM EDTA decreased the CFU by 3 or 4.5 logs, respectively. Remarkably, in the presence of 0.05 mM C12/C14/C15-EDTA under the same illumination, PpIX could even eradicate all the bacteria (reducing CFU by more than 8 logs).

Another important finding is the critical role of the alkyl chain length on the sensitizing activity of aEDTAs. After 2 h illumination and at 0.05 mM aEDTA, the CFU reduction by PpIX was in the following order: C14-EDTA > C12-EDTA ~ C15-EDTA > C8-EDTA ~ C16-EDTA ~ C18-EDTA >  > EDTA. Importantly, C14-EDTA facilitated the complete elimination of all bacteria within a 2 h illumination period, whereas C12-EDTA and C15-EDTA required 3 h of illumination to achieve the bacterium eradication.

Similar sensitizing effects of EDTA or aEDTAs (C12/C14/C15) were observed (Additional file 1: Figures S8, S9) for PpIX at 8.89 or 177.73 μM, albeit to a lesser extent. Other studies reported that 25 μM PpIX alone could inactivate 3 logs of *S. aureus* at 1 × 10^7^ CFU mL^−1^ [29]; but in this work, 8.89 μM PpIX with 0.05 μM C14-EDTA led to an 8-log reduction in CFU even at 100 times higher bacteria density.

We further benchmarked the antimicrobial efficacy of PpIX/C14-EDTA with Photogem, a hematoporphyrin derivative used successfully in clinical cases such as skin cancer and bacterial inactivation. Photogem was reported to reduce *S. aureus* CFU by less than 7 logs at 20 μM and a light fluence of 60 J cm^−2^. [30] In contrast, here we showed that the combination of 17.77 μM of PpIX and 0.05 mM of C14-EDTA completely eradicated the same amount of *S. aureus* (> 8 logs) at the same light fluence. These results demonstrate that combining PpIX with aEDTAs, particularly C14-EDTA, enhanced photodynamic activity against *S. aureus*, surpassing the other compounds, such as Photogem.

### Biofilm eradication by PpIX/C14-EDTA

**Fig. 3:**
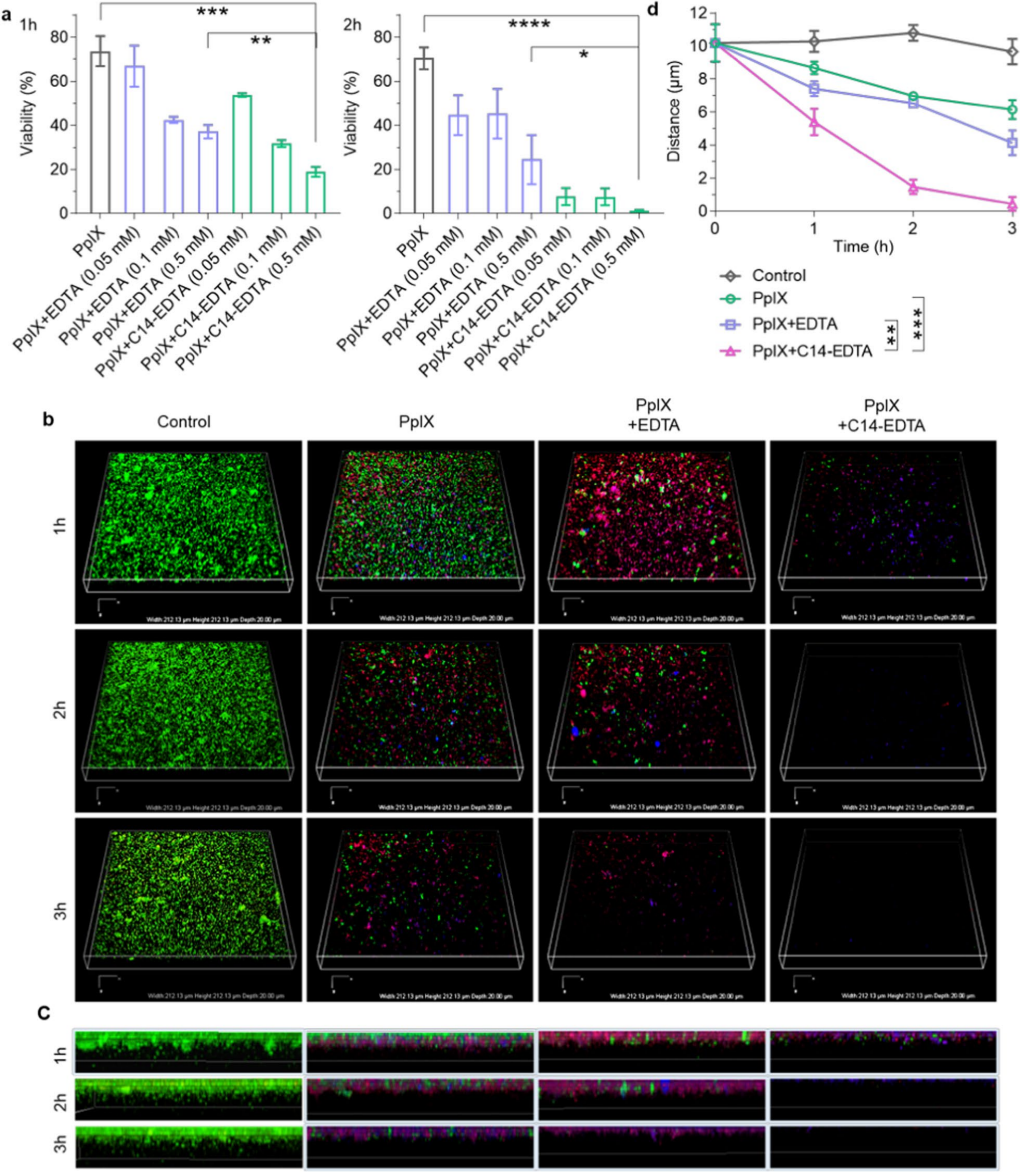
*S. aureus* biofilm eradication assay. **a** Mature *S. aureus* biofilms were treated with the combinations of PpIX (17.77 μM) and EDTA or C14-EDTA (0.05 mM, 0.1 mM, or 0.5 mM), followed by illumination for 1 h or 2 h; the remaining biofilms were subjected to the crystal violet assay to determine the viability. **b**–**c** 3D reconstruction **b** and cross-sections **c** of *S. aureus* biofilms by CLSM imaging; the biofilms were treated with 17.77 μM of PpIX and 0.1 mM of EDTA or C14-EDTA and then illuminated for the indicated durations; the biofilms were then stained using the Live/Dead BacLight^™^ Bacterial Viability Kit and observed using CLSM; Green: Syto9, Red: PI, Blue: PpIX. **d** The biofilm thickness quantified by Image J in **c**. Data are presented as mean ± SD (*n* = 3). ****P < 0.0001, ***P < 0.001, **P < 0.01

The excellent PDT activity of PpIX/C14-EDTA combination motivated us to investigate its biofilm eradication capacity. Biofilms are known to be highly resistant against antibiotics and other antimicrobial agents [31], and thus require significantly higher doses for effective eradication than planktonic bacteria [32]. We observed that treating the biofilm with 17.77 μM of PpIX for 1 h illumination only decreased the bacterial viability by 27%; PpIX combined with 0.05 or 0.5 mM of EDTA reduced bacterium population by 33% or 63%, respectively (Fig. 3a). In contrast, the combination of PpIX with 0.05 or 0.5 mM of C14-EDTA led to 47% and 81% of biofilm eradication. Prolonging the illumination time to 2 h increased the biofilm eradication effect; PpIX (17.77 μM) with 0.05 mM C14-EDTA eradicated over 90% of the biofilms.

We further employed confocal laser scanning microscopy (CLSM) to visualize the biofilm eradication process (Fig. 3b). The biofilms were treated with PpIX (17.77 μM) or its combination with EDTA (0.1 mM) or C14-EDTA (0.1 mM) and illuminated for 1, 2, or 3 h, respectively. The biofilms were then stained using the Live/Dead BacLight™ Bacterial Viability Kit, which includes Syto9 (a fluorescent dye that probes the total bacteria populations) and PI (a membrane-impermeable nuclear staining dye). After 1 h illumination, most bacteria in the PpIX-treated biofilm remained alive (green) and there were still bacteria alive even after 3 h illumination. EDTA did enhance the PpIX bacteria-killing. Interestingly, biofilms treated with both PpIX and PpIX with EDTA exhibited reduced green signals after 2 or 3 hours of illumination but retained a detectable signal even after 2 hours of illumination. This demonstrates that PpIX, whether used alone or in conjunction with EDTA, can effectively eliminate bacteria within the biofilm, yet falls short of complete biofilm removal. Significantly, the biofilm treated with the combination of PpIX and C14-EDTA showed a reduced green signal after 1 hour of illumination, and the signal vanished completely after 2 hours, indicating its potent ability to eradicate the biofilm. Quantitatively, after 3 h illumination, the biofilms treated with PpIX or PpIX/EDTA had thicknesses of about 6.1 μm and 4.2 μm, respectively, while that treated with PpIX/C14-EDTA was barely measureable, only about 0.4 μm thick (Fig. 3c and d). This indicates that PpIX/C14-EDTA effectively eradicated the biofilms.

### PDT activity of PpIX/C14-EDTA combinations in the infected ulcers

Next, we used an animal model of infected ulcers to assess the therapeutic effect of the PpIX and its C14-EDTA combination. The ulcers were created using an 8 mm diameter punch and locally infected with *S. aureus*. The ulcer sites were treated with different combinations and subjected to a light fluence of 60 J cm^−2^. Considering that ulcer lesions secrete various proteins, cytokines, and other components that may inactivate the treatment agents, we increased the concentrations of PpIX and EDTA/C14-EDTA to 177.73 μM and 0.5 mM, respectively.

**Fig. 4:**
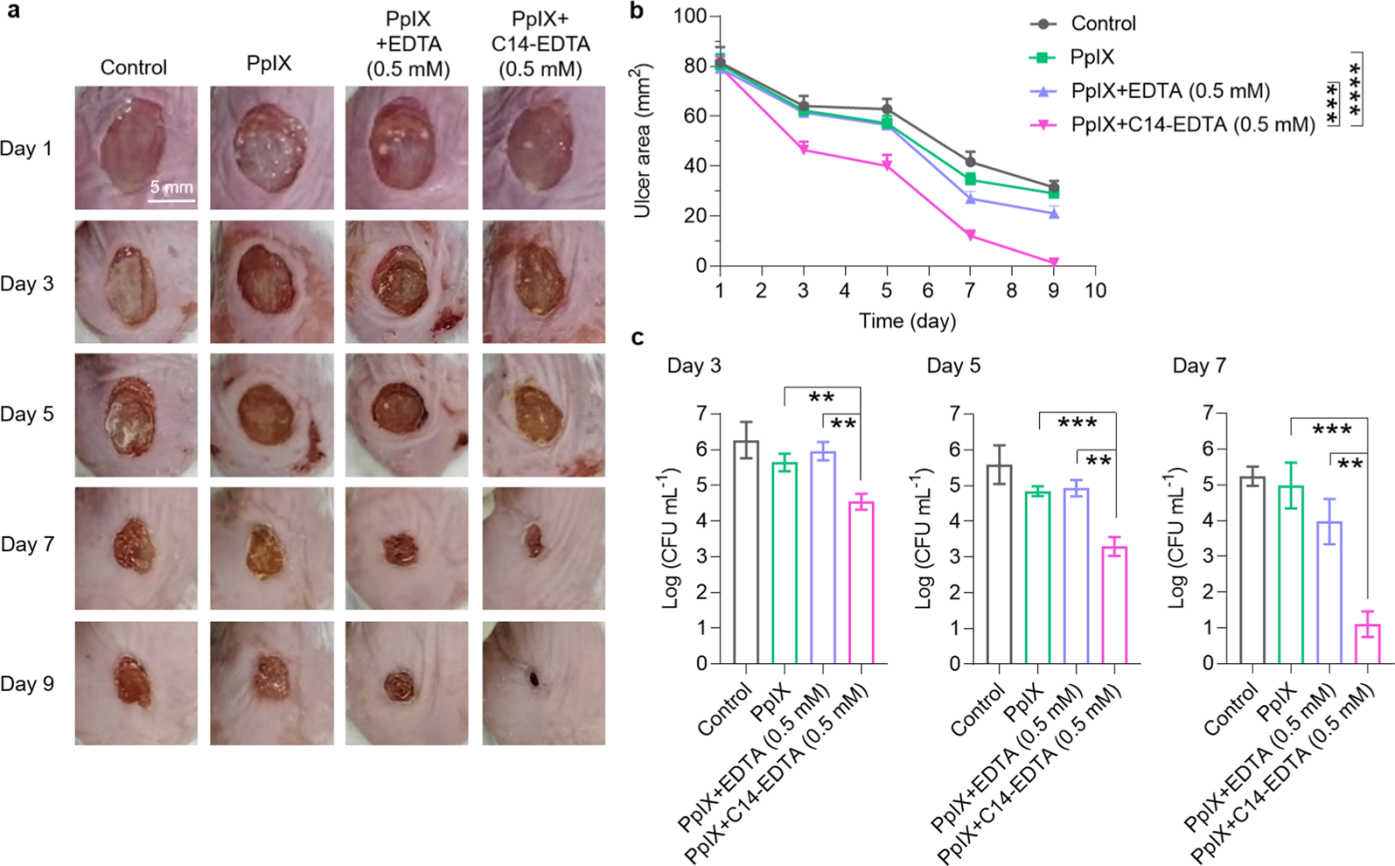
In vivo therapeutic effects of PpIX combinations on an animal model of infected ulcers. **a** The images of the ulcers after different treatments. **b** Average ulcer area of each group as a function of time. **c** The amount of bacteria collected at the ulcer lesions after different treatments for 3, 5, or 7 days. Data are presented as mean ± SD (*n* = 3). ****P < 0.0001, ***P < 0.001, **P < 0.01

Figure 4 shows that PpIX treatment failed to promote wound healing, having the same size of the ulcer areas as control even after 9 days (Fig. 4a). The combination of PpIX/EDTA reduced the ulcer area to ~ 20 mm^2^ on Day 9. PpIX/C14-EDTA showed the most remarkable therapeutic efficacy, which almost completely healed the ulcers, leaving only 1.5 mm^2^ ulcer area (Fig. 4b). The treatment effectiveness was further quantitated by a plate count assay. The PpIX/C14-EDTA treatment consistently and significantly outperformed other treatments in efficiently reducing the bacterial population at all time-points. It reduced the *S. aureus* CFU by 5 logs on Day 7 (Fig. 4c). We also evaluated the wound healing effects of PpIX combining other aEDTA. The results indicated that C14-EDTA outperformed its counterparts not only in terms of antimicrobial ability but also in wound healing capacity and bacteria elimination at the ulcer site (Additional file 1: Figure S12, S13). These findings highlight the significant therapeutic potential of PpIX/C14-EDTA in treating infected ulcers in vivo for the clinical management of infected ulcers.

### Mechanism investigation

**Fig. 5:**
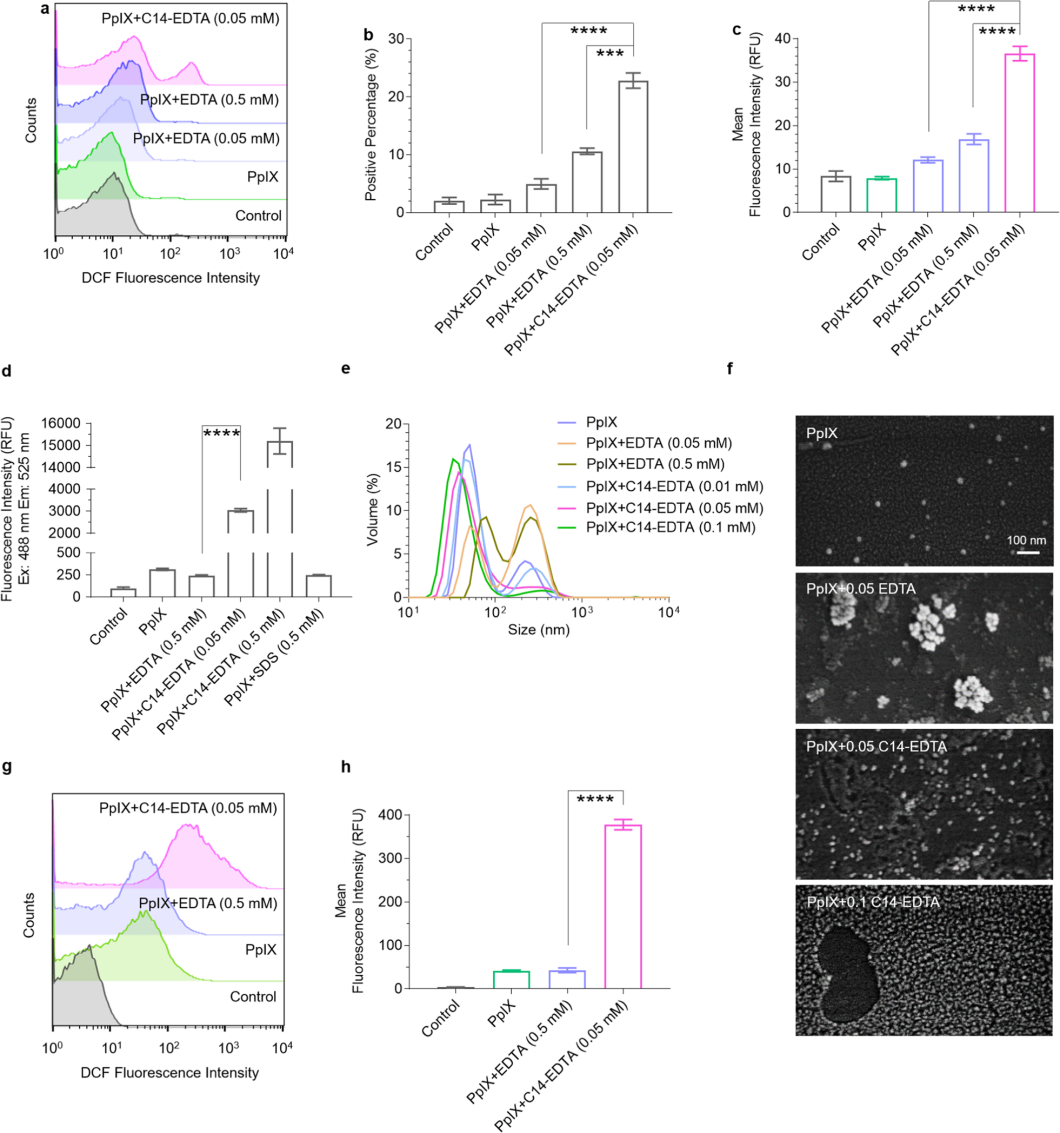
Mechanism investigation of PpIX and C14-EDTA in antimicrobial. **a** Flow cytometry analysis of the ROS levels in *S. aureus* after different treatments. The bacteria suspension was incubated with PpIX (17.77 μM) or its combination with EDTA or C14-EDTA for 1 h, the DCFH-DA probe for 15 min, and then irradiated for 30 min. **b**–**c** Percentage **b** and mean fluorescence intensity **c** of ROS-positive cells in panel *a*. **d** ROS detection generated by PpIX (17.77 μM) or its combinations in aqueous solution in the absence of bacteria. DCFH-DA was added into each well and incubated for 15 min. After illumination for 30 min, the fluorescence intensity was detected by spectrophotometer. **e**–**f** Size distribution measured by DLS **e** and SEM images **f** of PpIX or its combinations after 1-h incubation (PpIX:17.77 μM, EDTA/C14-EDTA: 0.05 mM). **g**, **h** PpIX accumulation in *S. aureus* in terms of counts **g** or intensity **h**. Bacterium suspension was pre-incubated with PpIX (17.77 μM) or its combination with EDTA or C14-EDTA for 1 h, followed by 1 h illumination. The accumulation percentage was then measured by flow cytometry (Ex: 488 nm; Em: 525 nm). Data are presented as mean ± SD (*n* = 3). ****P < 0.0001, ***P < 0.001, **P < 0.01

We next set out to understand how C14-EDTA enhances the antibacterial photodynamic activity of PpIX. We first used DCFH-DA to probe the ROS levels in differently treated bacteria via flow cytometry. As expected, the combination of PpIX (17.77 μM) and C14-EDTA (0.05 mM) induced more bacteria (23%) to generate ROS. In contrast, when treated with PpIX alone (17.77 μM) or its combinations with EDTA (0.05 or 0.5 mM), only 5% and 10% of the bacteria had detectable ROS generation (Fig. 5a, b). A similar trend was also observed in terms of the mean fluorescence intensity for each group (Fig. 5c).

Next, we investigated how C14-EDTA promoted ROS production by PpIX. We found that in the absence of bacteria, C14-EDTA itself substantially enhanced the photo-activated ROS production of PpIX (Fig. 5d). As the ROS production of PpIX can mirror the aggregation degree, we inferred that the presence of C14-EDTA might alleviate the aggregation of PpIX, which was confirmed by DLS and SEM results (Fig. 5e, f).

Further, C14-EDTA significantly facilitated the accumulation of PpIX in bacteria. Flow cytometry analysis demonstrated that bacteria treated with 17.77 μM of PpIX exhibited a mean fluorescence intensity of 40 RFU (relative fluorescence units), while those treated with PpIX/C14-EDTA (0.05 mM) had a corresponding value of 380 RFU (Fig. 5g, h). In contrast, EDTA only mildly enhanced the antibacterial activity of PpIX and had little effect on singlet oxygen production, regardless of the presence of bacteria. Additionally, EDTA did not reduce the aggregation of PpIX or increase its accumulation in bacteria. Moreover, a common surfactant, sodium dodecyl sulfate (SDS), did not reduce the aggregation of PpIX, either. These results demonstrate that both the EDTA and alkyl chain structures are crucial for C14-EDTA to mitigate PpIX aggregation.

We also investigated whether the intrinsic metal chelation of EDTA impacts C14-EDTA’s ROS-enhancing effect. It turned out that the enhancement of the antibacterial effect was essentially abolished, once barring the interaction between EDTA and bacteria by conjugating EDTA to Wang resin or blocking EDTA’s chelating ability using calcium ions (Additional file 1: Figure S10). Collectively, these results depict a potential mechanism of action of C14-EDTA, in which C14-EDTA alleviates the aggregation of PpIX, increases the bacterial membrane permeability, and sequesters the divalent cations such as Ca^2+^ (Additional file 1: Figure S11), jointly promoting the accumulation of PpIX in bacteria as well as the PDT activity.

**Fig. 6:**
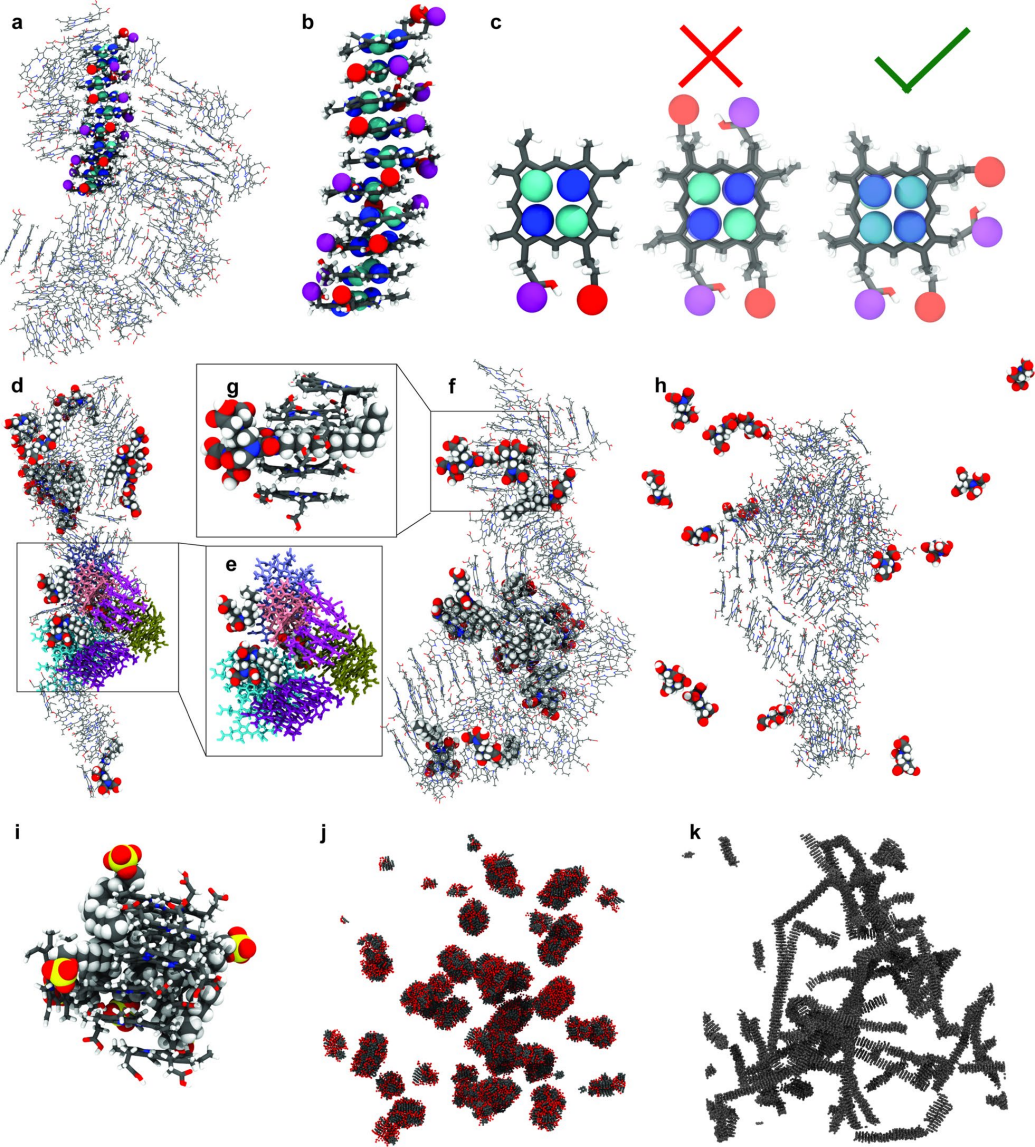
Molecular Dynamics simulations. **a**, **b** Cluster formation of PpIX in solution. Clusters are comprised of stacked PpIX molecules forming rod-like structures. An isolated stack is depicted in **b**. **c** The organization of PpIX in these rods occurs in defined pattern. Neighboring molecules are either 90 degree or 270  rotated against each other while a 0 or 180 rotation is not permitted. **d**, **e** C8-EDTA interacts with the cluster but assembles on the surface of the PpIX cluster. No C8-EDTA was found to penetrate the stacked molecules as shown in **e**. **f** C14-EDTA can penetrate in-between the layers of the PpIX stacks. **g** Zoomed version of the C14-EDTA cluster where the hydrophobic tail penetrates in-between the neighboring PpIX molecules. **h** EDTA does not penetrate between the layers of the PpIX stacks. (i) SDS forms a co-existing cluster that is attached to PpIX. **j** The Martini simulations with 2000 PpIX molecules show the formation of large, nanometer-sized clusters. **k** Small aggregates form in the presence of C14-EDTA

We performed MD simulations of the interactions between PpIX, C8-EDTA and C14-EDTA to interrogate how C8-EDTA and C14-EDTA alleviates the aggregation of PpIX. The results indicated that PpIX does form clusters in solution, in agreement with the DLS and SEM results. These clusters were found to exhibit an almost crystalline structure, in which the planar faces of PpIX align in a rod-like structure with few defects in the particle (Fig. 6a, b). Importantly, the PpIX molecules can only align in certain rotations at 90  or 270 degrees against the neighboring molecule (Fig. 6c). Both C8-EDTA and C14-EDTA were found to interact with the PpIX clusters. While C8-EDTA was observed to attach to the surface of the clusters (Fig. 6d, e), C14-EDTA was found to penetrate and disrupt the crystalline structure (Fig. 6f, g). The disruption of PpIX clusters is critical to form smaller particles and appears a unique effect of the synthesized structure of EDTA with an alkyl chain. EDTA alone does not interact with the PpIX cluster as evident in Fig. 6h. SDS, in comparison, forms a co-existing cluster that is attached to PpIX (Fig. 6i). However, the crystalline structure is not affected.

While all-atom simulations provide atomistic detail and accuracy, modern computers are unable to simulate cluster sizes that are comparable with the experimental length scales. We thus performed coarse grained simulations to study the macroscopic dynamics. PpIX cluster formation was also observed in these larger coarse-grained MD simulations (Fig. 6j, k). These simulations show that the PpIX clusters form through aggregation of the crystalline rods that were already observed in the all-atom simulation. Importantly, C14-EDTA also interacts with the clusters and disrupts the crystalline structure resulting in smaller, spherical particles that are surrounded by C14-EDTA.

## Conclusion

In summary, our study has demonstrated that C14-EDTA is a potent enhancer for PpIX-mediated PDT activity. C14-EDTA showed remarkable efficacy in eliminating not only planktonic *S. aureus* but also highly resistant *S. aureus* biofilms, even at relatively low concentrations when combined with PpIX. Mechanism investigations revealed that C14-EDTA could disrupt PpIX aggregation by interfering with PpIX crystallization. Furthermore, C14-EDTA substantially increased PpIX accumulation within bacteria, thereby enhancing the PDT activity. We envision that this straightforward and effective approach could be extended for various antimicrobial applications, especially against the notoriously drug-resistant Gram-positive bacteria biofilms.

### Supplementary Information

**Additional file 1: Figure S1.** Molecular mass determination of 2a: calculated for C_18_H_33_N_3_O_7_: 403.23; found: 402.20 [M-H]^-^. **Figure S2.** Molecular mass determination of 2b: calculated for C_22_H_41_N_3_O_7_: 459.29; found: 458.30 [M-H]^-^. **Figure S3. **Molecular mass determination of 2c: calculated for C_24_H_45_N_3_O_7_: 487.33; found: 486.30 [M-H]^-^. **Figure S4.** Molecular mass determination of 2 day: calculated for C_25_H_47_N_3_O_7_: 501.34; found: 500.40 [M-H]^-^. **Figure S5.** Molecular mass determination of 2e: calculated for C_26_H_49_N_3_O_7_: 515.36; found: 514.40 [M-H]^-^. **Figure S6.** Molecular mass determination of 2e: calculated for C_28_H_53_N_3_O_7_: 543.39; found: 542.40 [M-H]^-^. **Figure S7.** The wavelength spectrum of the LED illuminator. **Figure S8.** aEDTA improved PDT efficacy of PpIX (8.89 μM) in eliminating planktonic *S. aureus*. **Figure S9.** aEDTA improved PDT efficacy of PpIX (177.73 μM) in eliminating planktonic *S. aureus*. **Figure S10.** Bacterial viability after different treatments. **Figure S11.** ICP (Inductive Coupled Plasma Emission Spectrometer) analyzing the element content of Wang-EDTA after culturing with *S. aureus* overnight. **Figure S12.** In vivo therapeutic effects of PpIX (177.73 μM) with a series of different chain lengths of alkylated EDTA combinations on the wound healing model. **Figure S13.** Bacteria amount at the ulcer lesions after different treatments (PpIX: 177.73 μM) evaluated by the plate counting assay. **Figure S14.** The bond length and bond angle distributions of the final model. **Figure S15.** The force-field parameter of the all-atom and coarse –grained models.

## Data Availability

The datasets collected and/or analyzed during the current study available from the corresponding author on reasonable request.
