# Supplementary material for: Alkylated EDTA potentiates antibacterial photodynamic activity of protoporphyrin
Source: J Nanobiotechnology. 2024 Apr 8;22:161. doi: 10.1186/s12951-024-02353-3 (PMC11003131; doi:10.1186/s12951-024-02353-3)
Supplement: Supplementary file 1 — Additional file 1: Figure S1. Molecular mass determination of 2a: calculated for C18H33N3O7: 403.23; found: 402.20 [M-H]-. Figure S2. Molecular mass determination of 2b: calculated for C22H41N3O7: 459.29; found: 458.30 [M-H]-. Figure S3. Molecular mass determination of 2c: calculated for C24H45N3O7: 487.33; found: 486.30 [M-H]-. Figure S4. Molecular mass determination of 2 day: calculated for C25H47N3O7: 501.34; found: 500.40 [M-H]-. Figure S5. Molecular mass determination of 2e: calculated for C26H49N3O7: 515.36; found: 514.40 [M-H]-. Figure S6. Molecular mass determination of 2e: calculated for C28H53N3O7: 543.39; found: 542.40 [M-H]-. Figure S7. The wavelength spectrum of the LED illuminator. Figure S8. aEDTA improved PDT efficacy of PpIX (8.89 μM) in eliminating planktonic S. aureus. Figure S9. aEDTA improved PDT efficacy of PpIX (177.73 μM) in eliminating planktonic S. aureus. Figure S10. Bacterial viability after different treatments. Figure S11. ICP (Inductive Coupled Plasma Emission Spectrometer) analyzing the element content of Wang-EDTA after culturing with S. aureus overnight. Figure S12. In vivo therapeutic effects of PpIX (177.73 μM) with a series of different chain lengths of alkylated EDTA combinations on the wound healing model. Figure S13. Bacteria amount at the ulcer lesions after different treatments (PpIX: 177.73 μM) evaluated by the plate counting assay. Figure S14. The bond length and bond angle distributions of the final model. Figure S15. The force-field parameter of the all-atom and coarse –grained models. [file 12951_2024_2353_MOESM1_ESM.docx]

**Supporting information to:**

**Alkylated EDTA Potentiates** **Antibacterial Photodynamic Activity of Protoporphyrin**

Ying Piao^1^, Sebastian Himbert^2*^, Zifan Li^1^, Jun Liu^3^, Zhihao Zhao^1^, Huahai Yu^1^, Shuangshuang Liu^1^, Shiqun Shao^1*^, Michael Fefer^3^, Maikel C. Rheinstädter^2*^, Youqing Shen^1*^

^1^ Zhejiang Key Laboratory of Smart Biomaterials and Key Laboratory of Biomass Chemical Engineering of Ministry of Education, College of Chemical and Biological Engineering, Zhejiang University, Hangzhou 310058, China.

^2^ Department of Physics and Astronomy, McMaster University, Hamilton ON, L8S 3Z5, Canada

3 Suncor AgroScience, Mississauga, ON, L5K 1A8, Canada

* Corresponding authors: himberts@mcmaster.ca, shenyq@zju.edu.cn; rheinstadter@mcmaster.ca; shiqun.shao@zju.edu.cn;


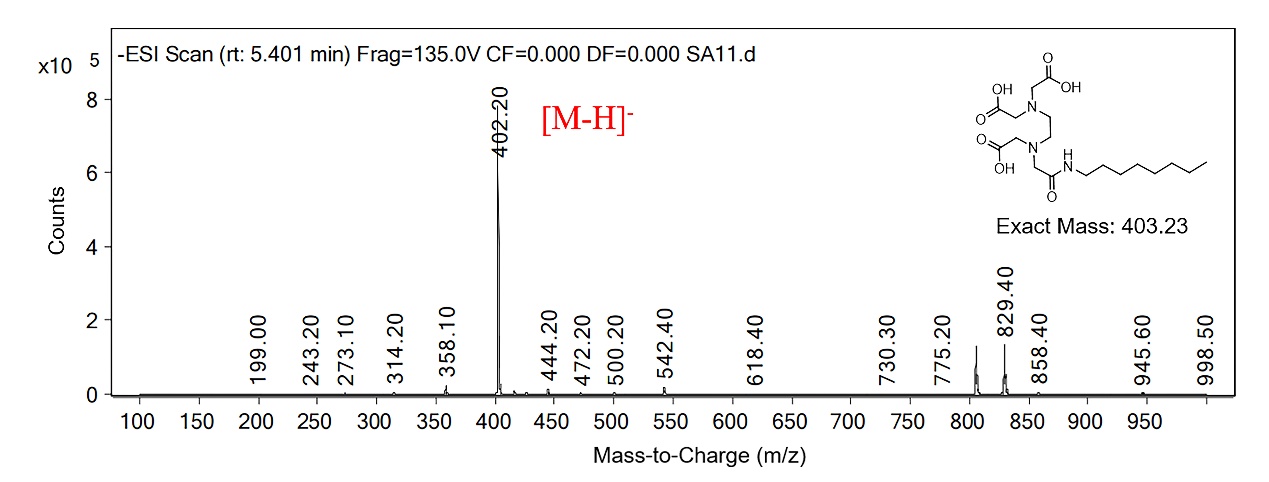


**Figure S1**. **Molecular mass determination of 2a: calculated for C_18_H_33_N_3_O_7_: 403.23; found: 402.20 [M-H]^-^.**


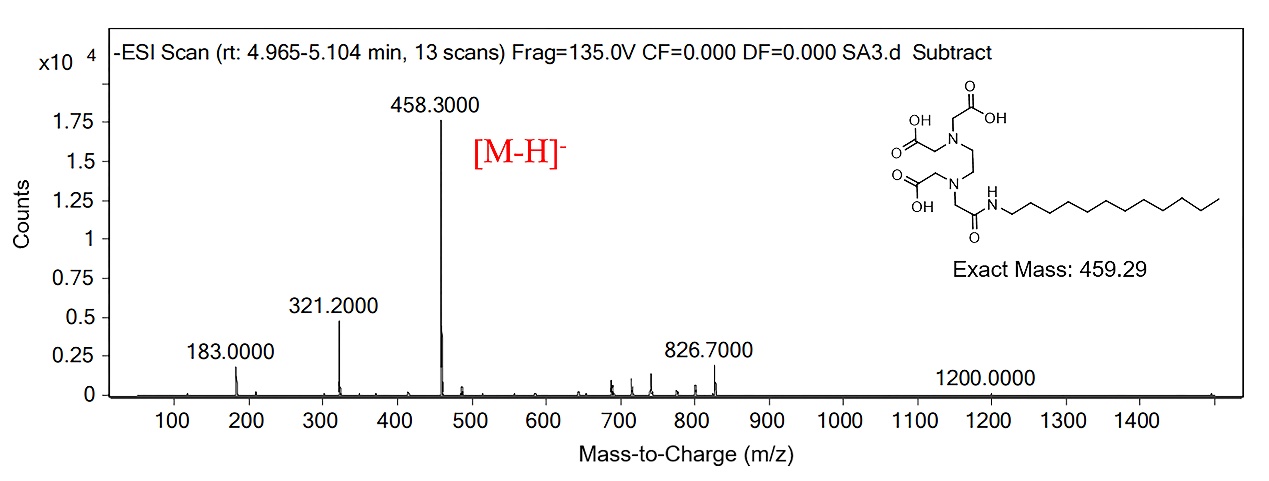


**Figure S2**. **Molecular mass determination of 2b: calculated for C_22_H_41_N_3_O_7_: 459.29; found: 458.30 [M-H]^-^.**

^
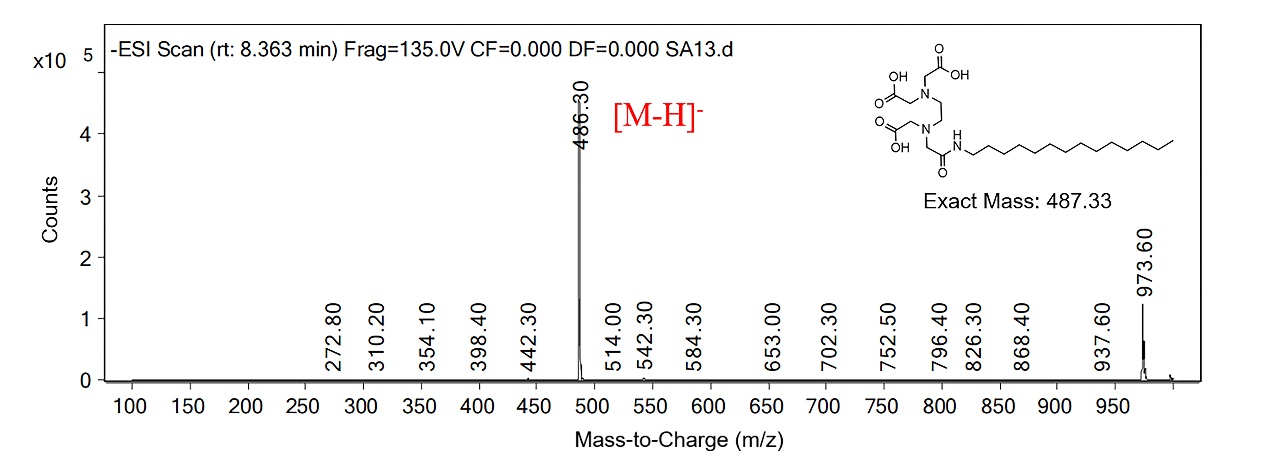
^

**Figure S3**. **Molecular mass determination of 2c: calculated for C_24_H_45_N_3_O_7_: 487.33; found: 486.30 [M-H]^-^.**

^
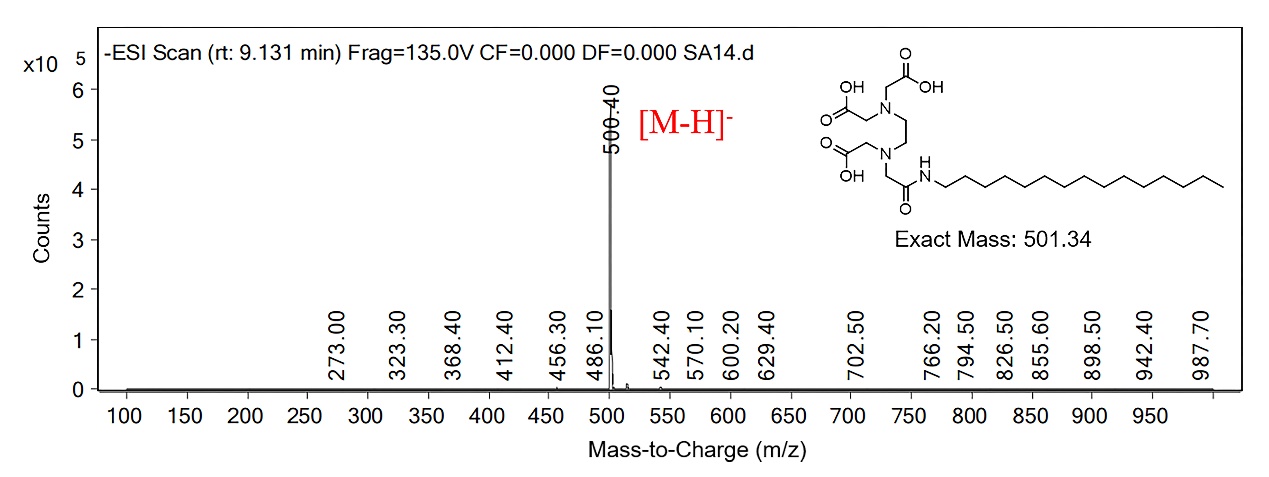
^

**Figure S4**. **Molecular mass determination of 2d: calculated for C_25_H_47_N_3_O_7_: 501.34; found: 500.40 [M-H]^-^.**

^
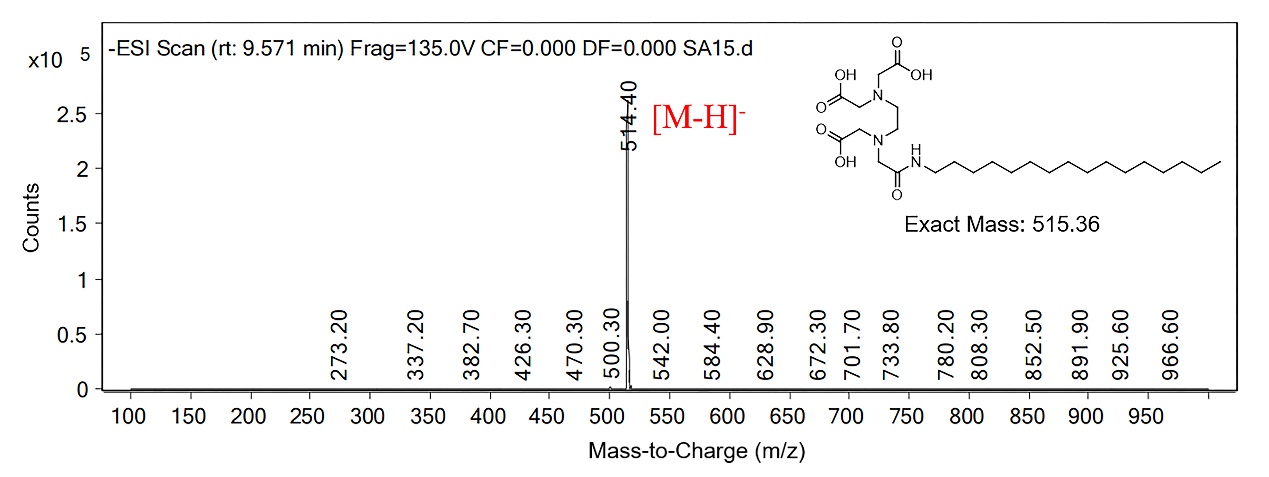
^

**Figure S5**. **Molecular mass determination of 2e: calculated for C_26_H_49_N_3_O_7_: 515.36; found: 514.40 [M-H]^-^.**

^
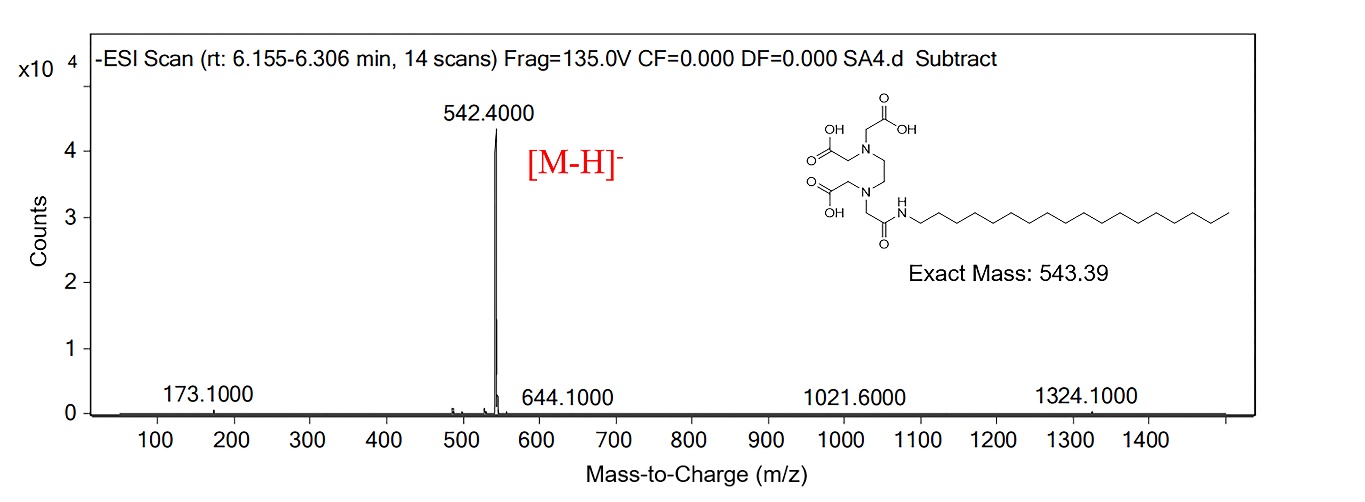
^

**Figure S6**. **Molecular mass determination of 2e: calculated for C_28_H_53_N_3_O_7_: 543.39; found: 542.40 [M-H]^-^.**

**
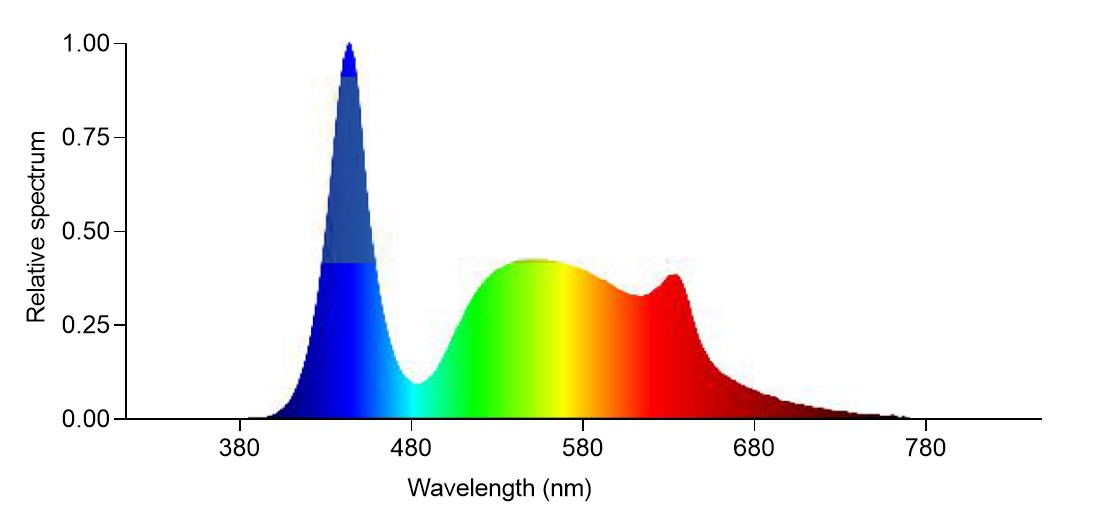
**

**Figure S7**. **The wavelength spectrum of the LED illuminator.**


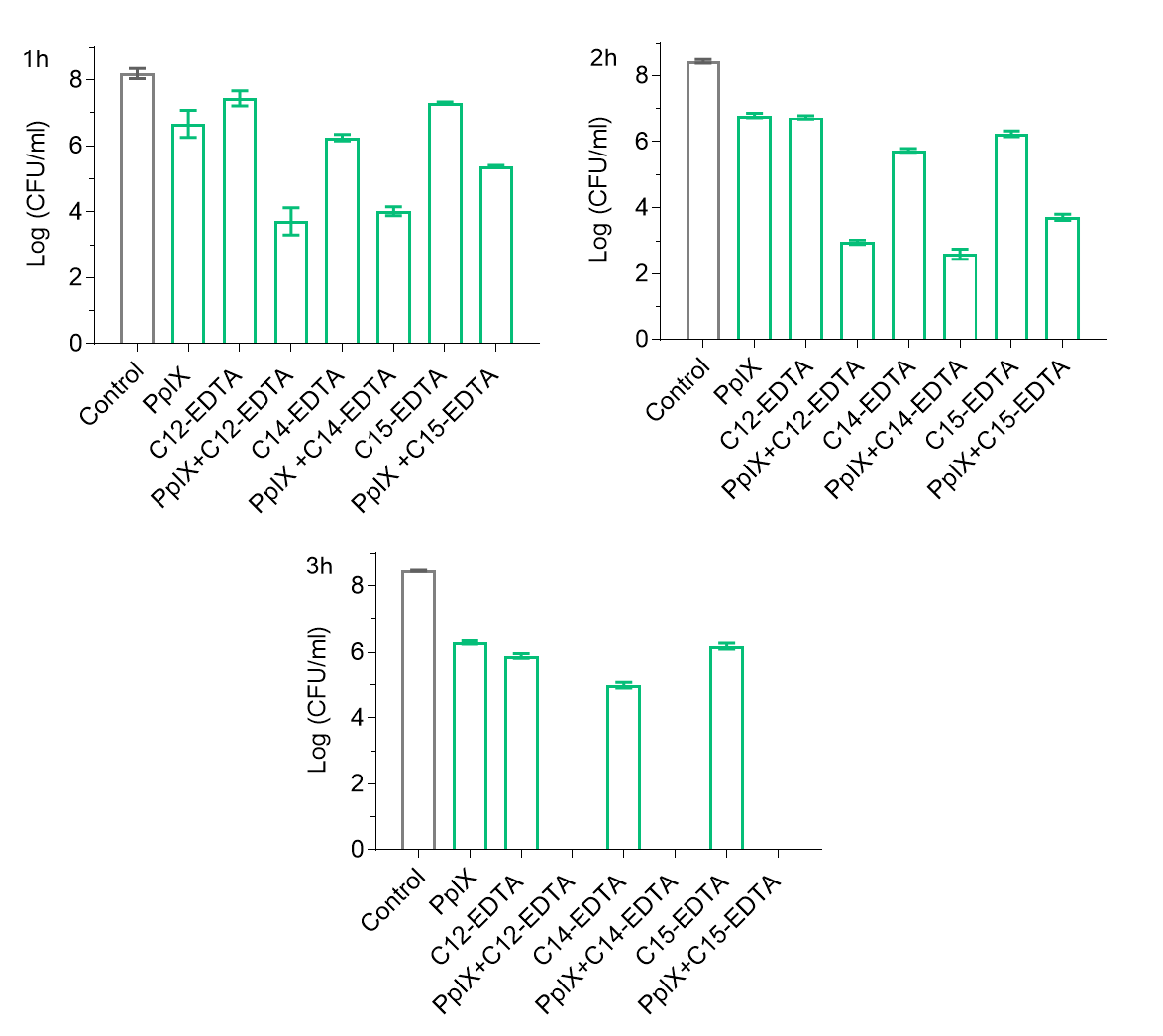


**Figure S8**. **aEDTA improved PDT efficacy of PpIX (8.89 μM) in eliminating planktonic *S. aureus*.** The same procedures were conducted as that using 17.77 μM PpIX. Briefly, exponentially growing *S. aureus* of OD600 0.3 were treated with 8.89 μM of PpIX or PpIX-aEDTA combinations and the antibacterial activities were evaluated by the plate counting assay.


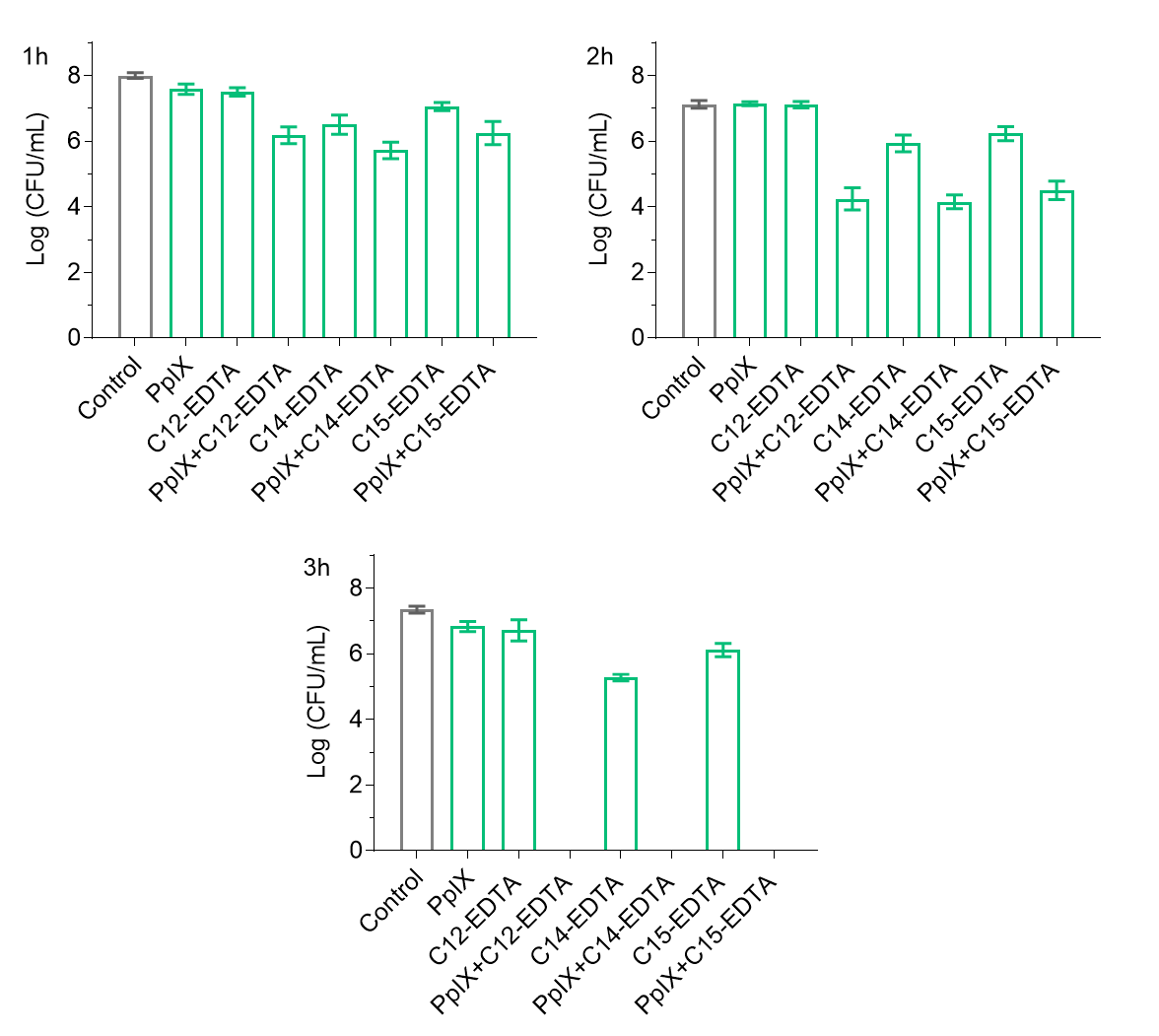


**Figure S9**. **aEDTA improved PDT efficacy of PpIX (177.73 μM) in eliminating planktonic *S. aureus***. The same procedures were conducted as that using 17.77 μM or 8.89 μM PpIX.


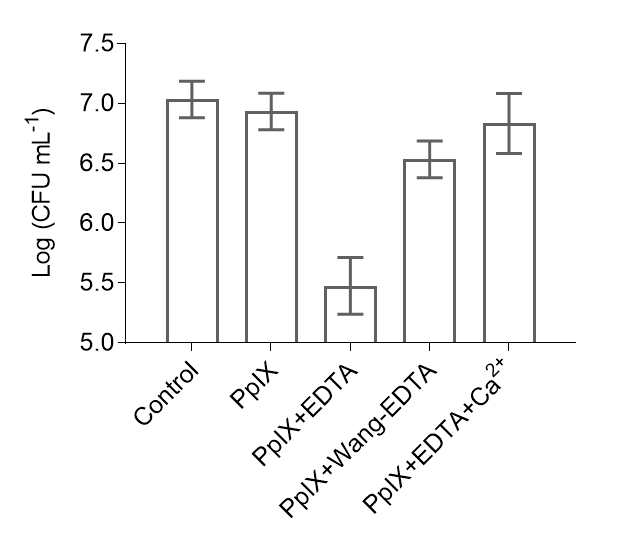


**Figure** **S10**. **Bacterial viability after different treatments**. *S. aureus* cells were treated with 177.73 μM PpIX or its combinations with EDTA (0.5 mM), Wang-EDTA (0.5 mM), or EDTA (0.5 mM) plus CaCl_2_ (0.5 mM) for 16 h. The CFU were counted by the plate counting method after 24-h incubation.


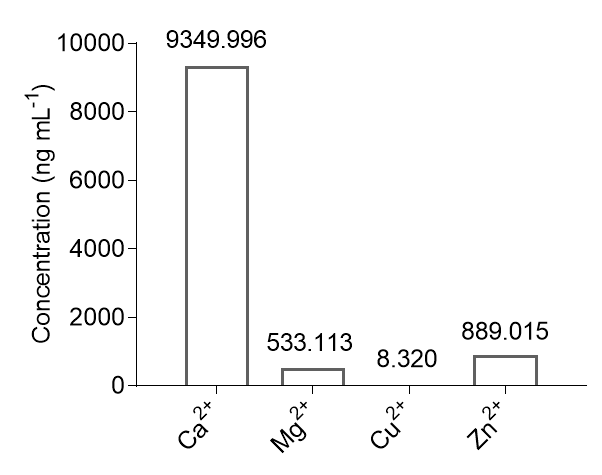


**Figure** **S11**. **ICP (Inductive Coupled Plasma Emission Spectrometer) analyzing the element content of Wang-EDTA after culturing with *S. aureus* overnight.**


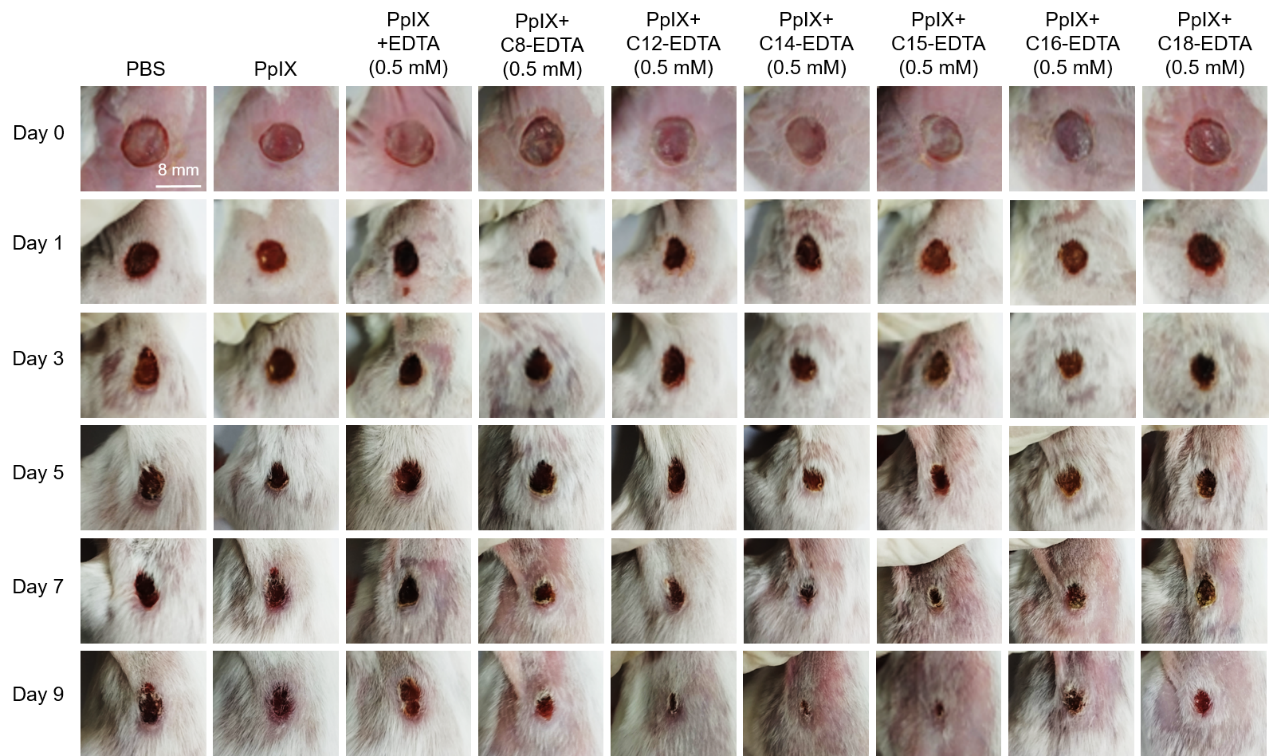


**Figure S12**. ***In vivo* therapeutic effects of PpIX (177.73 μM) with a series of different chain lengths of alkylated EDTA combinations on the wound healing model.**


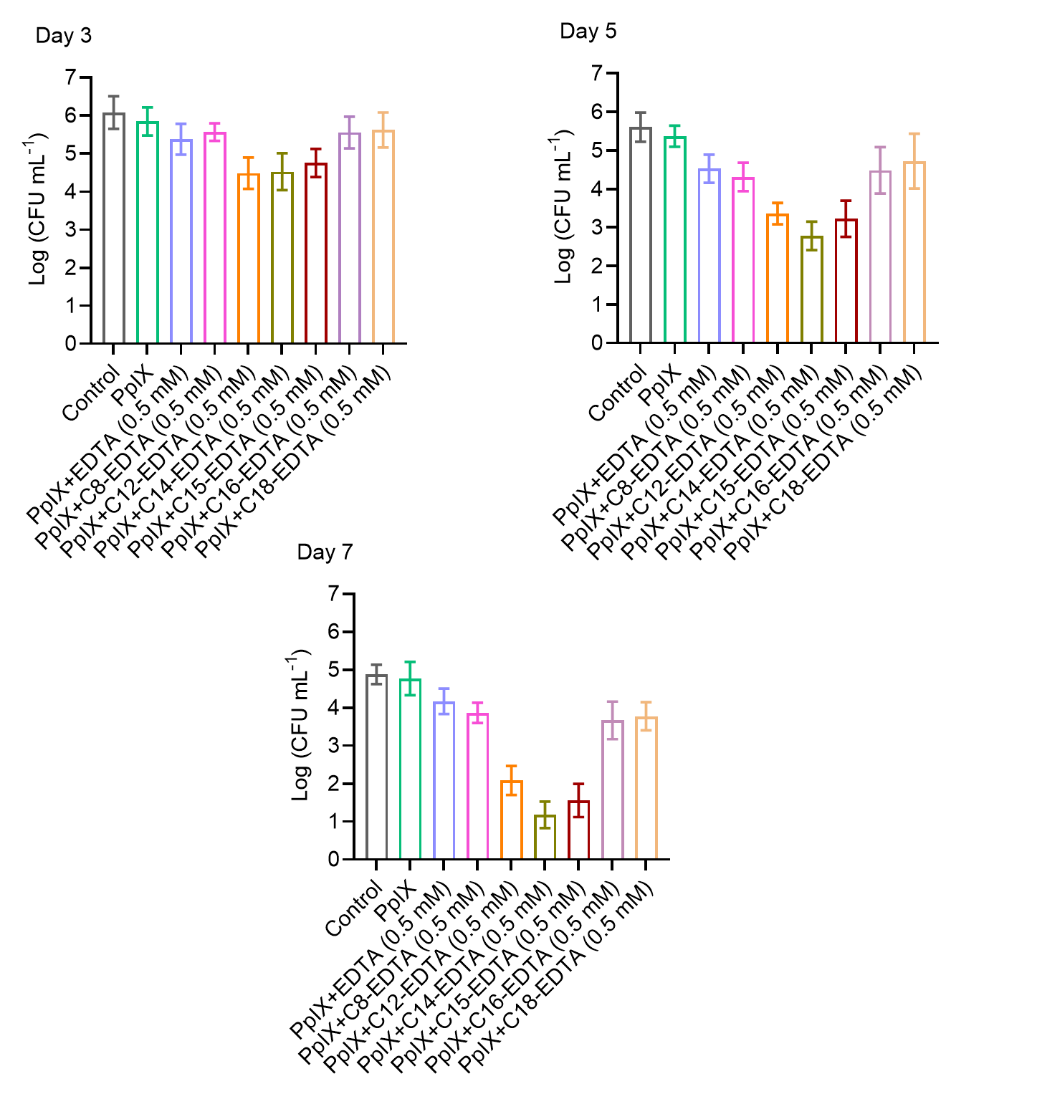


**Figure S13**. **Bacteria amount at the ulcer lesions after different treatments**

**(PpIX: 177.73 μM) evaluated by the plate counting assay.**


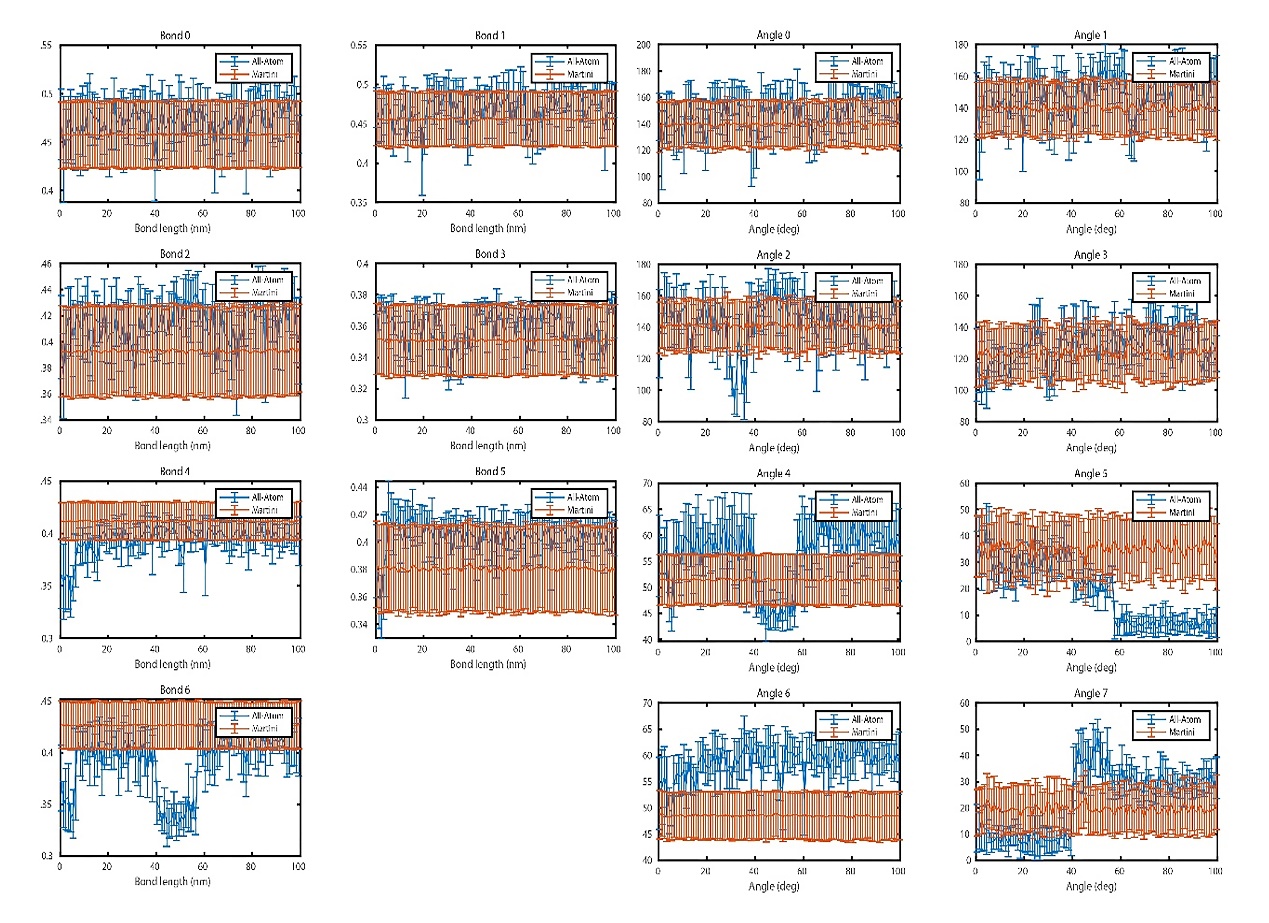


**Figure S14**. **The bond length and bond angle distributions of the final model.**


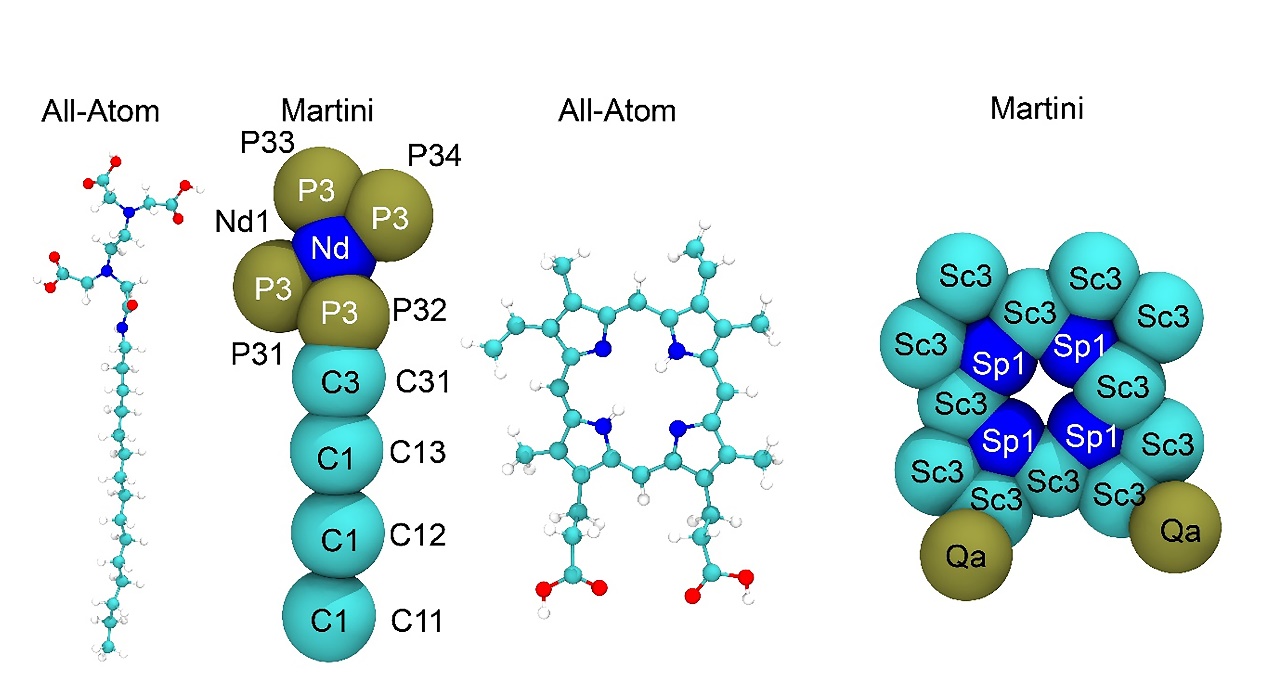


**Figure S15**. **The force-field parameter of the all-atom and coarse –grained models.**
